# Ageism in the time of COVID-19

**DOI:** 10.1177/1368430220983452

**Published:** 2021-03-04

**Authors:** Hannah J. Swift, Alison L. Chasteen

**Affiliations:** 1 University of Kent, UK; 2 University of Toronto, Canada

**Keywords:** ageism, age stereotypes, COVID-19, intergenerational relations

## Abstract

In this article, we outline how the response to the coronavirus (COVID-19) pandemic has the potential to fundamentally change how we think and feel about our own age, and how we think and feel about other age groups. Specifically, we outline how discourse surrounding the pandemic has strengthened the homogeneous view of older adults as vulnerable, has socially stigmatized being an older adult, and has exacerbated hostile and benevolent expressions of ageism. We explore the impact of these changing dynamics on intergenerational cohesion and relations, and propose that understanding theories of ageism will be essential for how we handle future pandemics in order to reduce the potential negative impact of crises on individuals as well as on communities and societies.

Research exploring the nature and consequences of ageism, directed towards and experienced by older adults, has been growing in traction in the last few years due to increasing recognition of population ageing, workforce ageing, and the need to enhance and not hinder older adults’ participation and contribution to society. However, the global COVID-19 outbreak has the potential to exacerbate expressions and experiences of ageism in Western cultures. We illustrate how the discourse surrounding the pandemic has strengthened the homogeneous view of older adults as vulnerable, has socially stigmatized being an older adult, and has increased hostile and benevolent ageism. We then explore the potential impact on intergenerational relations and set out new avenues for future research.

## Vulnerability Narrative

At the beginning of March 2020, when the spread of COVID-19 was escalating, the media across Europe and the Americas consistently paired the terms “vulnerable” and “older people” when describing the pandemic (Ayalon et al., 2020). Although well intentioned, this messaging had two very damaging consequences. First, it strengthened the homogeneous view of older adults as vulnerable, an already widely embedded negative age stereotype (Cohn-Schwartz & Ayalon, 2020; Swift et al., 2019). This view can be damaging to future older adults. As posited by stereotype embodiment theory (SET) and the risks of ageism model (RAM), if future generations internalize negative age stereotypes, it can result in self-limiting views of old age, which can be a risk to health, well-being, and active participation in later life (Levy, 2009; Swift et al., 2017). The vulnerability narrative is also concordant with negative age stereotypes of incompetence, which underpin age discrimination in the workplace, in health and social care, and in the provision of goods and services (Levy et al., 2020; Swift et al., 2017). Age stereotypes can also inform the ways we interact with older people, such as using overly patronizing gestures or tones (Chasteen et al., 2020; see also Fisher & Ryan, 2021, for a discussion of gender stereotypes during COVID-19). This is costly not only to individuals’ health and well-being (Jackson et al., 2019), but also to the economy. The cost of age discrimination to the annual production of goods and services in the US is estimated to be $850 billion (AARP, 2020). Another study estimates that the US loses $63 billion annually due to health costs related to ageism (Levy et al., 2020).

Second, the messaging suggests that only older people should be worried about or are at risk of contracting the virus and, by implication, that young people are not vulnerable or, worse, are resilient to it (Gerontological Society of America [GSA], 2020). In many countries, the framing of the pandemic failed to point out that people of all ages are vulnerable to COVID-19. A few months in, data suggest that adults in their 20s and 30s are the largest proportion of COVID-19 carriers (Henley, 2020), with many suffering serious health consequences as a result of the virus. The framing of vulnerability is important because it influences perceived risk to COVID-19, which in turn influences health behaviours and the spread of the virus.

Together, the representation of older adults as vulnerable and those who are younger as invulnerable has potential to cause friction between generations by influencing how we think age groups should behave in order to control the virus. For example, the suggestion that only older adults should isolate can be discriminatory by suppressing another reality, which is the diversity of this age group spanning some 40 to 50 years. Scholars argue it would “deprive society of many people who are productive and active and who can be a key part of the solution by supporting the economy, families and communities” (Stafford, 2020). The vulnerability narrative also misrepresents age as the primary indicator of risk when, in reality, other factors are actually more important, such as the nature and seriousness of preexisting health conditions (Ayalon et al., 2020; GSA, 2020). Therefore, the vulnerability narrative is problematic because it homogenizes older adults, it can create tensions between generations by influencing how we think age groups should behave during the pandemic, and it disregards the contribution of older adults to society.

## Stigmatizing Social Identity

COVID-19 has been represented as a disease that only affects older people thus increasing the stigmatization of being an older person (Aronson, 2020). This perception—coupled with the vulnerability narrative and increased pairing of the terms “elderly” and “older adults” with death, mortality, and morbidity—has consequences for how we feel about our age and the ageing process. According to terror management theory, one explanation for ageism is that it stems from people’s fears of death and dying, which contribute to fears about being old, being perceived as old, and having a stigmatized social identity (Martens et al., 2005; see also Esses & Hamilton, 2021, for discussion of disease avoidance and anti-immigrant attitudes during COVID-10). As a consequence, we tend to distance ourselves from identifying and engaging with older adults in order to protect ourselves from the discomfort associated with mortality salience (Martens et al., 2005). This can manifest as a disconnect between people’s chronological age and their self-identification as an older person—or the age they feel, known as subjective age (Weiss & Lang, 2012).

Emerging evidence suggests that the pandemic seems to be exacerbating this disconnect. A three-wave study in the US with participants aged 18 and over (*N* = 3,738) revealed that participants’ tendency to feel younger increased with the emergence of the pandemic, and was predicted by the extent to which participants believed COVID-19 to be a threat to older adults (Terracciano et al., 2020). This is indicative of a strategy to protect the self from negative information, such as stereotypes about ageing (Weiss & Lang, 2012) and the threat of COVID-19, by feeling younger (Terracciano et al., 2020). For older adults, psychologically distancing themselves from their age could protect them from threats to their identity, but it could also mean they do not recognize themselves in the descriptive language used by the media or policymakers, because the descriptions do not conform to how they view themselves (see Kruglanski et al., 2021, for discussion of threats to self during COVID-19). This, in turn, could influence engagement in preventative behaviours to reduce the transmission of COVID-19 if they are not deemed relevant to the self.

The additional subjectivity surrounding the age at which we think someone becomes an older adult (Abrams et al., 2011) also means it is up to individuals to determine who these vulnerable “older people” are. As a consequence, people who did not consider themselves “old” before, or are psychologically distancing themselves from this age group, might not be able to escape being perceived as an older adult by others. Therefore, people may still find themselves subject to ageist comments or behaviours from friends, peers, colleagues, or strangers, even if they do not view themselves as “old.”

## Devaluing Perceived Social Status of Older Adults

In addition to stigmatizing old age, public discourse surrounding the pandemic has shed light on how societies value people’s lives and on the discrepancy between the value placed on older and younger people’s lives (Aronson, 2020). As highlighted by Fraser et al. (2020), the disregard for the impact of COVID-19 in care homes and the exclusion of nursing home residents from official death counts “could lead the public to conclude that their deaths were insignificant and to be expected (p. 693)” At the same time, the widespread use of the hashtag “BoomerRemover” (Aronson, 2020; Monahan et al., 2020) signals a lack of concern over how COVID-19 is affecting older generations. Xiang et al.’s (2020) analysis of posts on Twitter revealed that 1 in 10 tweets implied that the lives of older adults are less valuable, and downplayed the pandemic because it mostly affects older adults. In addition, the high mortality rates amongst older adults are considered inevitable or a normal outcome, worthy of jokes or ridicule, which is consistent with past findings showing the deaths of younger individuals are seen as more unjust than those of older people (Chasteen & Madey, 2003).

The devaluing of older adults’ lives can also be seen in narratives surrounding the distribution of resources needed to fight the virus, such as age-based rationing of ventilators and hospital beds (White & Lo, 2020). The increased demand for health care set against limited resources can lead people to justify the distribution of resources in favor of those who are younger, which implies that older people’s lives are less valuable.

## Hostile, Calculated Ageism

Devaluing of older adults’ social status in society is also present in expressions of “calculated ageism.” Calculated ageism refers to expressions or sentiments that justify prejudicial beliefs or unfair treatment with positive claims (Barrett et al., 2020). During the pandemic, public discourse that suggests older generations should sacrifice their lives in order to reduce the economic cost of COVID-19 for future generations has emerged (Barrett et al., 2020). In this case, the ageist expression is that older lives are expendable, which is set against the benefit of reduced economic burden on younger generations. This is a stark and clear expression of age bias against older adults, and a damaging characterization of how a society feels about its older generations (Aronson, 2020).

Although expressions of hostile, calculated ageism have arisen during the pandemic, they are not necessarily widely endorsed. Barrett et al. (2020) used thematic analysis to examine 188 tweets in response to Texas Governor Dan Patrick’s (aged 69) statement that encouraged self-sacrifice of those aged 70 plus for the economic benefit of America’s future generations. Only 5% of responses endorsed the statement, 90% opposed, and the remaining 5% conveyed no position. Support for calculated ageism centered on individual responsibility and patriotism. Opposition centered on its immorality, political-economic critiques (such as privileging the economic interest of the powerful few over the many), affirmations of older adults’ contribution and value, and the public health argument that outlined the importance of prioritizing preventative strategies. This example illustrates how COVID-19 has emboldened politicians and others to express hostile, ageist statements that were much less likely to be communicated before the pandemic.

## Benevolent, Compassionate Ageism

The distinction between hostile and benevolent expressions and experiences of ageism is well evidenced, with benevolent ageism characterized as subtler (Abrams et al., 2011; Chasteen et al., 2020). Benevolent ageism reflects the widely recognized view that there are mixed stereotypes of older adults, who are typically viewed negatively as incompetent, but positively as warm and friendly (Cuddy et al., 2007). This mixed view underpins feelings of pity, and drives paternalism, the need to protect, and actions of nurturing and helping (Cuddy et al., 2007). The pandemic, which exacerbated the vulnerability narrative, has conflated chronological age with impairment, incompetence, but also helplessness. These perceptions can increase feelings of pity and, therefore, also helping behaviours (Cuddy et al., 2007). Indeed, there has been an increase in social movements driven by social media to help and support people impacted by the virus (Vervaecke & Meisner, 2020).

Although well intentioned, these social movements can be a vehicle for benevolent ageism. Old age stereotypes can inform a biased view that older adults are the ones in more need of help and assistance (GSA, 2020). As such, older adults could find themselves on the receiving end of unwanted help and unwanted expressions of concern. Research has shown that unwanted patronizing expressions of concern are viewed as more acceptable when coming from family or friends compared with strangers (Horhota et al., 2019). It may be the case that the COVID-19 outbreak has only heightened such patronizing treatment of older people, given the perceived acceptability that existed prior to the pandemic.

Social care movements also need to be careful not to reinforce the vulnerability narrative and impressions of older adults as warm but incompetent (Vervaecke & Meisner, 2020). The internalization of these stereotypes could lead future older generations to behave in line with expectations of helplessness (Levy, 2009). They also have the potential to be problematic by promoting a dependence–support script that upholds youth-centered power and privilege as abled-bodied helpers of less privileged stereotyped groups (Vervaecke & Meisner, 2020).

## Implications for Intergenerational Relations

Relations between generations could be significantly disrupted by the pandemic. The vulnerability narrative serves to strengthen younger people’s already negative age stereotypes of need and dependency (Ayalon et al., 2020). In addition, motivation to maintain a positively distinct social identity could lead younger people to increase the psychological distance between themselves and older age groups, which have been stigmatized by the pandemic (Terracciano et al., 2020). This has the potential to increase ageist views and lead to misunderstandings as well as reduced empathy and perspective taking, which can disrupt intergenerational cohesion (Abrams et al., 2011).

Expressions of hostile ageism that devalue older adults’ social status and frame their consumption of resources as disproportionate or excessive, and as detrimental to younger generations, could be a source of conflict and create tension between generations. This narrative sees older adults as violating a prescriptive norm of consumption, which is the view that older adults should not consume resources reserved for future generations (North & Fiske, 2013). Violating this norm can result in younger adults holding more hostile attitudes towards their older counterparts (North & Fiske, 2013). In addition, narratives that highlight the negative impact of the pandemic reflected, for instance, on youth unemployment, which violates beliefs about succession of power and jobs to younger generations, could further exacerbate intergenerational tensions (North & Fiske, 2013). On the other hand, younger adults’ good intentions to help others during the pandemic could be rejected if they are deemed patronizing (Horhota et al., 2019; Vervaecke & Meisner, 2020).

Of course, older adults are not the only ones susceptible to be portrayed negatively by the media during the pandemic. A further dynamic that has potential to harm cohesion between generations is the representation of younger adults as reckless and irresponsible (Gharzai et al., 2020). This could result in hostile expressions of ageism towards younger adults (Chasteen et al., 2020), and in older adults resisting or avoiding contact with younger people.

## Future Directions and New Theoretical Challenges

SET and the RAM suggest the vulnerability narrative will be self-limiting to future generations and will negatively impact health, well-being, and longevity (Levy, 2009; Swift et al., 2017). A priority for researchers will be to monitor the extent to which negative age stereotypes are recognized and endorsed during and after the pandemic. The impact of psychological distancing from older age is likely to have both positive, such as persevering positive social identity (Weiss & Lang, 2012), and negative implications, such as disregarding self-relevant preventative information and be a barrier to intergenerational cohesion.

The double-edged nature of old age stereotypes is also reflected in hostile and benevolent expressions of ageism. Here, there is potential to test theoretical models—such as the stereotype content model (Fiske et al., 2002), the behavior from intergroup affect and stereotypes map (Cuddy et al., 2007), and prescriptive intergenerational-tensions (North & Fiske, 2013)—that outline connections between stereotypes, affect, and subsequent behaviours or expressions of prejudice. The emerging evidence suggests a rise in hostile and benevolent expressions, which should be monitored alongside the consequences on health, well-being, and how we think and feel about ageing and older people. Monahan et al. (2020) outline that positive responses to the pandemic that promote helping and prosociality can reinforce the value of older adults, with benefits to health and well-being, but that we should be aware they could also be a source of patronizing ageism, which is another avenue for research to explore.

Media reports that frame the pandemic as a burden on younger generations and suggest older adults violate norms of consumption and succession risk intergenerational cohesion by dismissing the contribution of older adults (North & Fiske, 2013). To prevent this, the heterogeneity and diversity of older adults should be recognized as well as the critical role they play in the response to and recovery from the pandemic (Kendall-Taylor et al., 2020). Testing these strategies to mitigate the potential negative impact of the pandemic could also be further explored. The impact of the media on attitudes and stereotypes regarding young people, and the consequences of this for expressions of ageism and intergenerational relations are also worthy of investigation, as it would help shape more holistic approaches and theories of ageism that span the life course. Policymakers should be cognizant of potential intergenerational divides that could be exposed by age-based policy placing restrictions on different age groups; also, the media could benefit from a code of conduct that includes age as a recognized protected characteristic.
